# PDE5 inhibition rescues mitochondrial dysfunction and angiogenic responses induced by Akt3 inhibition by promotion of PRC expression

**DOI:** 10.1074/jbc.RA120.013716

**Published:** 2021-01-13

**Authors:** Daniel G. Corum, Dorea P. Jenkins, James A. Heslop, Lacey M. Tallent, Gyda C. Beeson, Jeremy L. Barth, Rick G. Schnellmann, Robin C. Muise-Helmericks

**Affiliations:** 1 Department of Regenerative Medicine and Cell Biology, Medical University of South Carolina, Charleston, South Carolina; 2 Department of Pathology, Medical University of South Carolina, Charleston, South Carolina; 3 Department of Bioengineering, Duke University, Durham, North Carolina; 4 Department of Drug Discovery, Medical University of South Carolina, Charleston, South Carolina; 5 Department of Pharmacy, University of Arizona, Tucson, Arizona

**Keywords:** Akt3/PKBγ, angiogenesis, endothelial cells, mitochondria, peroxisome proliferator–activated receptor γ coactivator-1 α, PGC-1α, Akt PKB, PPARGC1A, phosphodiesterases, PRC, Akt3

## Abstract

Akt3 regulates mitochondrial content in endothelial cells through the inhibition of PGC-1α nuclear localization and is also required for angiogenesis. However, whether there is a direct link between mitochondrial function and angiogenesis is unknown. Here we show that Akt3 depletion in primary endothelial cells results in decreased uncoupled oxygen consumption, increased fission, decreased membrane potential, and increased expression of the mitochondria-specific protein chaperones, HSP60 and HSP10, suggesting that Akt3 is required for mitochondrial homeostasis. Direct inhibition of mitochondrial homeostasis by the model oxidant paraquat results in decreased angiogenesis, showing a direct link between angiogenesis and mitochondrial function. Next, in exploring functional links to PGC-1α, the master regulator of mitochondrial biogenesis, we searched for compounds that induce this process. We found that, sildenafil, a phosphodiesterase 5 inhibitor, induced mitochondrial biogenesis as measured by increased uncoupled oxygen consumption, mitochondrial DNA content, and voltage-dependent anion channel protein expression. Sildenafil rescued the effects on mitochondria by Akt3 depletion or pharmacological inhibition and promoted angiogenesis, further supporting that mitochondrial homeostasis is required for angiogenesis. Sildenafil also induces the expression of PGC-1 family member PRC and can compensate for PGC-1α activity during mitochondrial stress by an Akt3-independent mechanism. The induction of PRC by sildenafil depends upon cAMP and the transcription factor CREB. Thus, PRC can functionally substitute during Akt3 depletion for absent PGC-1α activity to restore mitochondrial homeostasis and promote angiogenesis. These findings show that mitochondrial homeostasis as controlled by the PGC family of transcriptional activators is required for angiogenic responses.

Endothelial cells (EC) rely mainly on glycolysis for energetic requirements and have a relatively low mitochondrial content that does not contribute to cellular ATP levels in a meaningful way (1). This contrasts with the high metabolic requirements of the heart and kidney, which require mitochondrial oxidative phosphorylation for ATP production. It is well-established that mitochondria perform functions independent of ATP generation, including the regulation of apoptosis, reactive oxygen (ROS) production, calcium signaling, and iron handling. Mitochondrial homeostasis is controlled by a balance of mitochondrial biogenesis, autophagy (turnover), and fission/fusion processes that maintain mitochondrial health (2, 3, 4). Increased mitochondrial biogenesis produces healthy mitochondria in times of increased energetic requirements and for the dilution of damaged mitochondria during times of stress. Fission and fusion events between mitochondria act to distribute undamaged mitochondrial components throughout the entire mitochondrial pool. For this balanced self-renewal to occur, mitochondria must constantly import and incorporate ∼1500 precursor proteins encoded in the nuclear genome into the mitochondria while simultaneously participating in fission and fusion events with other mitochondria (5). Active protein import machinery (translocase of the outer membrane and translocase of inner membrane complexes) (6) is required for the efficient import and appropriate folding of mitochondrial proteins. Under conditions of stress, such as oxidative stress or decreased mitochondrial protein import efficiency, the mitochondrial unfolded protein response (UPRmt) is initiated (2, 5). The UPRmt results in the expression of mitochondrial-specific chaperone proteins such as HSP60 and HSP10, and specific proteases (*e.g.* ClpP and Yme1L1) which attempt to refold or degrade damaged or aggregated proteins to restore mitochondrial proteostasis (7). Disruptions in mitochondrial homeostatic processes have been implicated in the vasculature, resulting in decreased endothelial barrier function and angiogenesis. These disruptions can lead to other pathologies including cardiovascular and neurodegenerative diseases.

We reported that serine/threonine kinase Akt3 is required for angiogenic responses, independent of Akt1 (8). Although both Akt family members are required to launch angiogenic responses, Akt3-null mice have an isoform-specific angiogenic deficit, suggesting that the roles of Akt1 and Akt3 are not redundant. Akt1 is specifically required for eNOS activation, an activity that cannot be compensated for by Akt3 (9, 10). Importantly, ECs recruited during an angiogenic challenge in Akt3-null mice had fewer mitochondria, a phenotype not shared with Akt1-null mice. Our work indicates that Akt3 modulates mitochondrial content in ECs downstream of vascular endothelial growth factor via regulation of both mitochondrial biogenesis and autophagy (8, 11), specifically by regulating the subcellular localization of peroxisome proliferator activated receptor γ coactivator 1-α (PGC-1α).

PGC-1α is a member of the PGC-1 family of transcriptional coactivators and includes PGC-1β and PGC-1–related coactivator (PRC). Although all three PGC-1 family members are known to regulate mitochondrial biogenesis, PGC-1α is known as the master regulator of mitochondrial biogenesis (12). PGC-1 is a target for numerous signaling pathways, including NO/cGMP (13, 14) β-adrenergic/cAMP through CREB (15) and calcium/calmodulin-dependent protein kinase (CaMK-IV) (16, 17). Most of these signaling pathways exert their effects on PGC-1α by directly affecting its expression, phosphorylation, and/or acetylation. However, Akt3 indirectly affects PGC-1α by controlling its nuclear retention via the regulation of CRM-1, the major nuclear export receptor thus increasing nuclear-encoded mitochondrial gene expression (8).

As a transcriptional co-activator, PGC-1α responds to physiological signals via its interactions with transcription factors such as NRF-1 and ERRα. PGC-1α and PGC-1β single knockout mice have relatively mild phenotypes that are exacerbated upon double knockout, indicating their functional redundancy (18). PGC-1β and PRC act similarly to PGC-1α in that they can also drive NRF-dependent gene expression. Blockade of PRC expression results in reduced metabolic capacity, decreased uncoupled respiration, and abnormal mitochondrial morphology (19). Recent findings suggest that PRC is responsive to mitochondrial and metabolic stress and may be linked to inflammation via its regulation of interleukin I (19, 20). Taken together, the PGC family of transcriptional co-activators is important for the regulation of mitochondrial biogenesis and mitochondrial homeostasis and shares some functional similarities.

Akt3 is required for both angiogenesis and overall mitochondrial content in ECs (8). Whether there is a direct link between mitochondrial homeostasis and angiogenesis is unknown. Given Akt3 regulates genes that control mitochondrial protein import and antioxidant defenses, we evaluated the role of Akt3 in the regulation of EC mitochondrial protein stress pathways. Here, we report that inhibition of Akt3 results in a decrease in mitochondrial homeostasis and increased expression of HSP10 and HSP60, suggesting an induction of UPRmt. We show that pharmacological induction of UPRmt by the model oxidant paraquat mimics Akt3 depletion, causing a reduction in mitochondrial homeostasis and a decrease in angiogenesis, linking mitochondrial homeostasis and angiogenesis. Using sildenafil, a phosphodiesterase 5 inhibitor, a potent inducer of mitochondrial biogenesis in renal proximal tubular cells and in mouse kidney via up-regulation of cGMP signaling (21), we sought to induce mitochondrial biogenesis following Akt3 knockdown to test whether angiogenic responses could be rescued. Our findings suggest that PRC expression is responsive to increased cAMP signaling and, that cAMP-dependent PRC expression can functionally substitute for PGC-1α under conditions of Akt3 depletion, demonstrating that mitochondrial homeostasis in human ECs is required for angiogenesis.

## Results

### Akt3 knockdown causes decreased maximal respiratory capacity in ECs mitochondria

**Figure 1:**
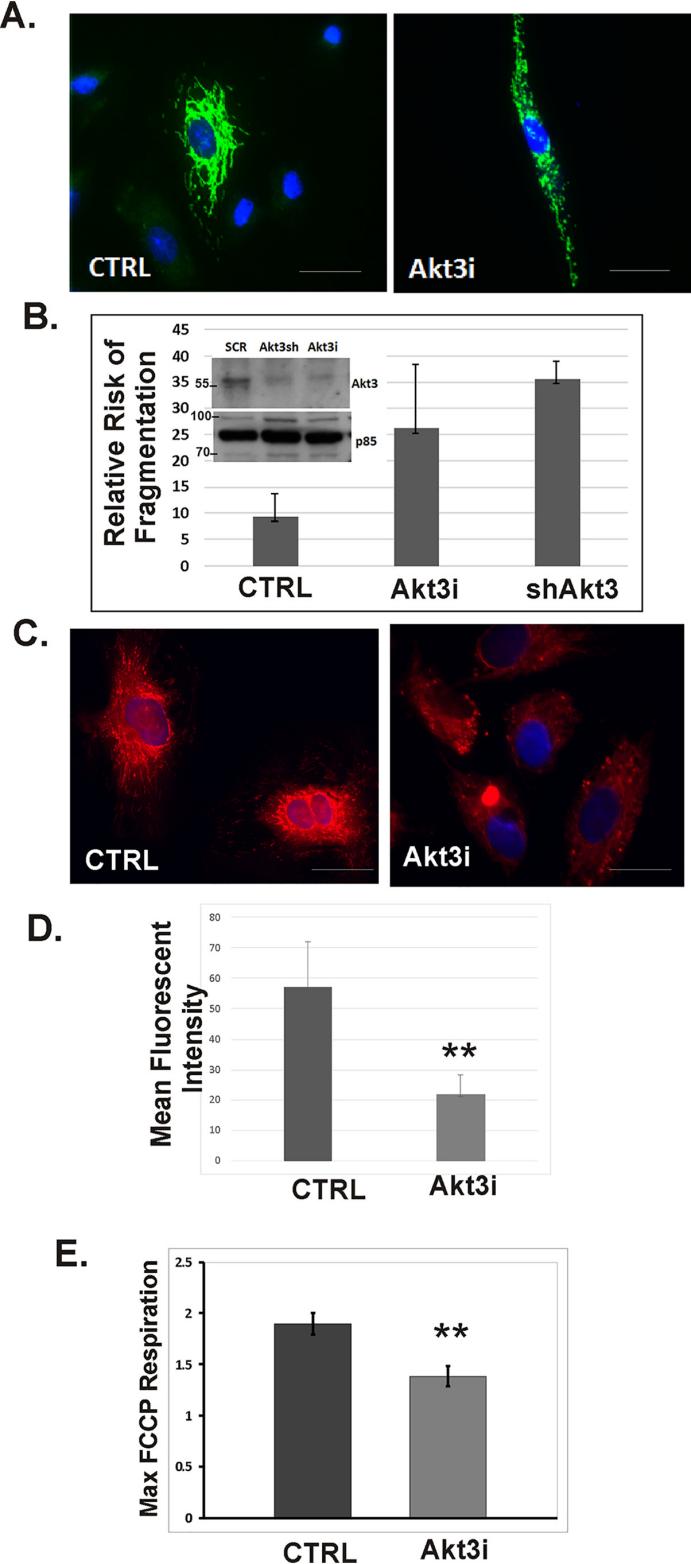
**Akt3 depletion results in reduced mitochondrial function.***A*, co-transfection of a mitochondrial-directed GFP plus an RNAi directed against Akt3 (*Akt3i*) or scrambled control were assessed for mitochondrial fragmentation by immunofluorescence. 40× images are shown. *Scale bar* indicates 400 μm. *B*, relative risk analysis fragmentation induced by Akt3 depletion using either RNAi or lentiviral transduction of an shRNA as compared with control. *Inset* shows an immunoblot of Akt3 knockdown under either RNAi or shRNA transduction. *C*, fluorescence microscopy images of ECs stained with MitoTracker Deep Red taken at equivalent exposure times. DAPI used as a nuclear stain. 40× images are shown. *Scale bar* indicates 400 μm. *D*, quantification of background-corrected MitoTracker Deep Red staining, shown as -fold change relative to SCR control. *E*, quantitation of maximal FCCP-uncoupled respiration of HUVECs following transfection with RNAi directed against Akt3 (*Akt3i*) or scramble (*SCR*) control using an Xf96 extracellular flux analyzer. **, *p* < 0.05 relative to respective control. *Error bars* indicate S.E.

Our studies have shown that Akt3 specifically controls mitochondrial biogenesis through its regulation of PGC-1α nuclear accumulation (8). Reduced Akt3 expression results in a decrease in mitochondrial biogenesis and an increase in autophagy (8). To test if Akt3 depletion also affected mitochondrial homeostasis, EC were transfected with Akt3 RNAi, transduced with an Akt3-specific shRNA or appropriate scrambled controls and assessed for mitochondrial fragmentation, mitochondrial membrane potential, and oxygen consumption rates in real time. As shown in Fig. 1*A*, Akt3 ablation results in an increased number of fragmented mitochondria as visualized using a mitochondrially directed GFP. Quantitation of relative risk of fragmentation (Fig. 1*B*) shows a 2.5 to 3.5 increased risk (siRNA *versus* shRNA) fragmentation as compared with control cells. The Western blotting in Fig. 1*B* shows Akt3 depletion with either siRNA or shRNA. Akt3 depletion also results in a decreased MitoTracker Deep Red uptake, a dye that requires membrane potential for its mitochondrial accumulation (Fig. 1*C*) resulting in a 3-fold decrease in fluorescent intensity in images with equivalent exposure times (Fig. 1*D*). Akt3 knockdown had no effect on the basal respiration of EC mitochondria (data not shown) but did cause an approximate 50% decrease in maximal respiratory capacity relative to scramble control following treatment with electron transport chain uncoupler FCCP (Fig. 1*E***).** These findings suggest that in addition to control of mitochondrial biogenesis, Akt3 also impinges on mitochondrial homeostatic mechanisms.

### Pharmacological induction of mitochondrial biogenesis in ECs

**Figure 2:**
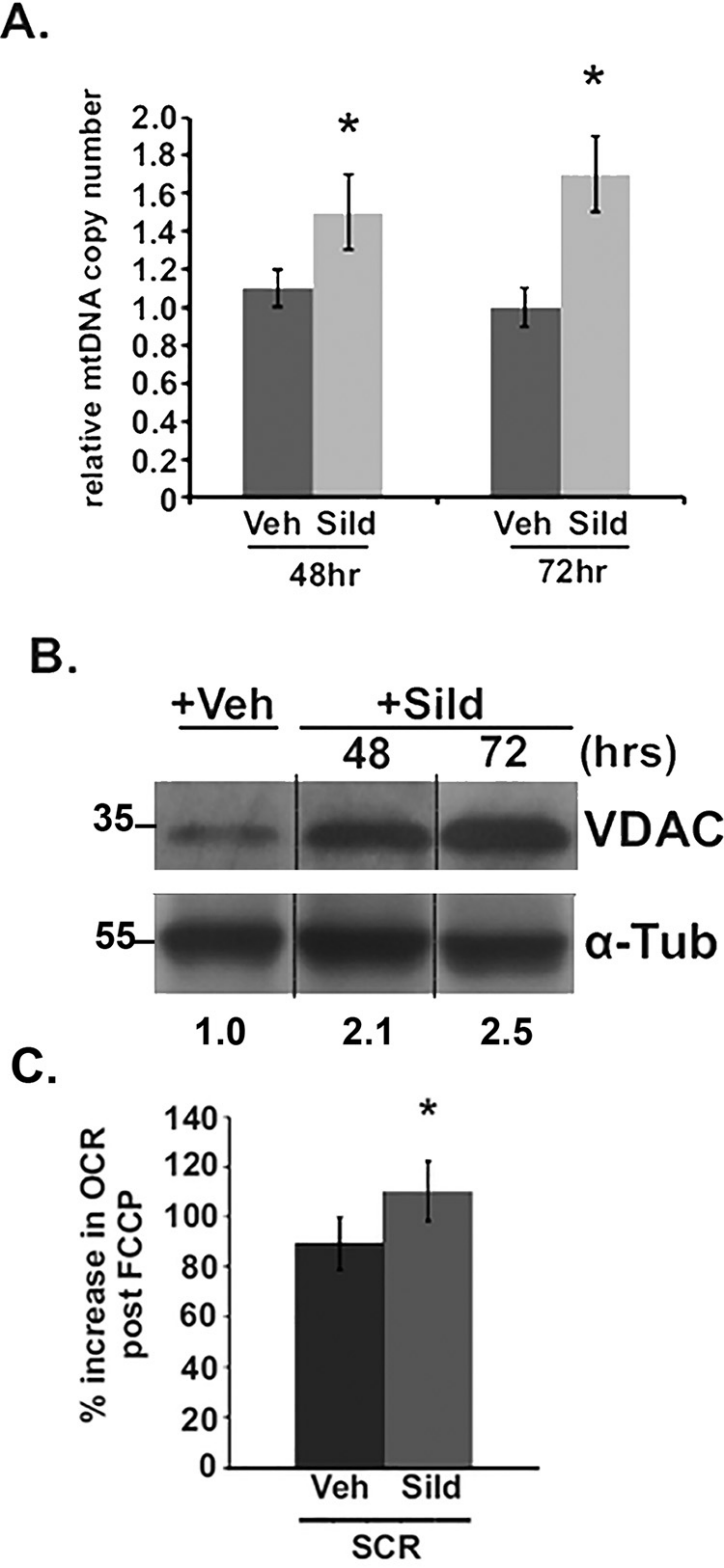
**Sildenafil increases mitochondrial biogenesis in ECs.***A*, analysis of mitochondrial DNA (mtDNA) copy number by real-time PCR in ECs treated with 100 nm sildenafil or vehicle for 48 or 72 h. *, *p* < 0.05. *B*, immunoblot analysis of VDAC protein expression in ECs treated with vehicle or 100 nm sildenafil for 48 or 72 h. α-Tubulin shown as loading control. *C*, analysis of basal respiration (*left*) and maximal FCCP-uncoupled respiration (*right*) in control ECs treated with vehicle or 100 nm sildenafil for 72 h. *, *p* < 0.05 relative to respective control. *Error bars* indicate S.E.

To examine other pathways that lead to mitochondrial biogenesis in endothelial cells we used the phosphodiesterase 5 inhibitor sildenafil, a potent inducer of mitochondrial biogenesis in renal proximal tubular cells and in mouse kidney (21), to induce mitochondrial biogenesis in HUVECs. ECs were treated with 100 nm sildenafil for 48 h and 72 h, and mitochondrial DNA (mtDNA) copy number was determined as a marker of mitochondrial biogenesis. Sildenafil increased mtDNA copy number at both 48 and 72 h of treatment (Fig. 2*A*). To confirm that sildenafil increased mitochondrial biogenesis, Western blot analyses for mitochondrial voltage-dependent anion channel (VDAC), a marker of mitochondrial protein content, were performed. VDAC protein was elevated at both time points (Fig. 2*B*), mirroring mtDNA copy number. In addition, respiratory analysis of ECs treated with 100 nm sildenafil for 72 h showed a 25% increase in maximal respiration following FCCP-induced uncoupling (Fig. 2*C*). Taken together, these data suggest that inhibition of PDE5 by sildenafil can induce mitochondrial biogenesis in ECs.

### Sildenafil rescues mitochondrial function and angiogenesis in ECs following Akt3 knockdown

**Figure 3:**
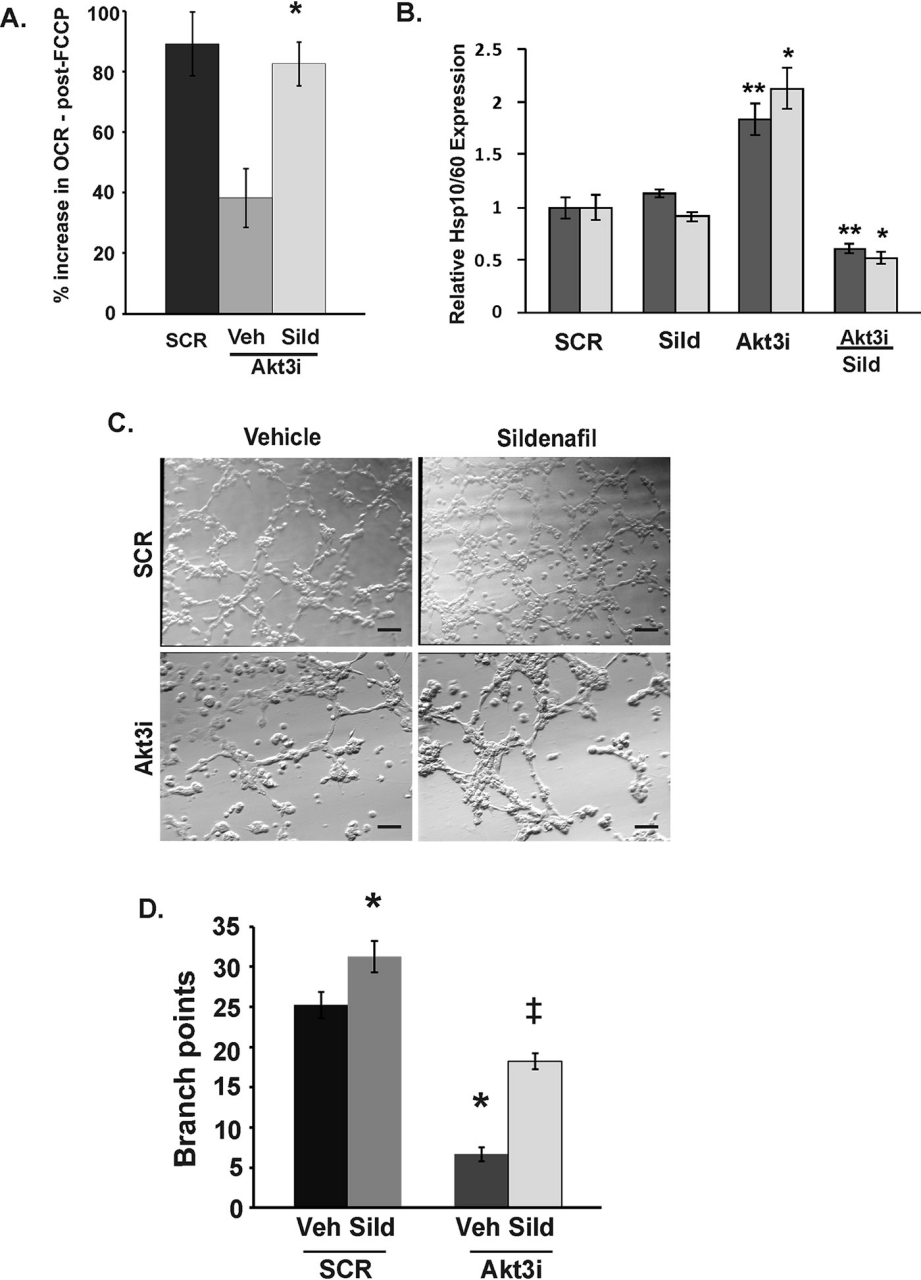
**Sildenafil rescues mitochondrial function and angiogenesis inhibited by Akt3 depletion.***A*, quantitation of maximal FCCP-uncoupled respiration of ECs following transfection with RNAi directed against Akt3 (*Akt3i*) or scramble (*SCR*) plus or minus the addition of sildenafil (100 μm) for 48 h using an Xf96 extracellular flux analyzer. *, *p* < 0.02; **, *p* < 0.05 relative to respective control. *B*, real-time PCR analysis of the relative expression of HSP60 and HSP10 expression following transfection of SCR or Akt3 RNAi and sildenafil plus or minus Akt3i transfection. Expression is relative to S26 as an internal control. *, *p* < 0.05; **, *p* < 0.03. *C*, HUVEC transfected with either scrambled control (*SCR*) or RNAi directed against Akt3 (*Akt3i*) treated with or without sildenafil (100 μm) prior to plating on Matrigel. *D*, quantitation of branch points in the angiogenesis assays described in (*C*) *p* values < 0.5. All *error bars* in indicate S.E. 10× magnifications are shown. *Scale bars* indicate 100 μm.

To determine whether treatment of ECs with sildenafil is sufficient to rescue mitochondrial dysfunction induced by Akt3 depletion, maximal respiration was measured in cells treated with sildenafil or vehicle at the 72 h following Akt3 knockdown. Akt3 knockdown decreased maximal respiratory capacity and sildenafil treatment restored maximal respiratory capacity in ECs (Fig. 3*A*). Next, we tested whether sildenafil treatment was sufficient to rescue Akt3-dependent mitochondrial chaperone expression. As shown in Fig. 3*B*, Akt3 depletion results in a 2-fold increase in the expression of HSP60 and HSP10, suggesting a role for Akt3 in the maintenance of mitochondrial homeostasis and control of the UPRmt. A 72-h treatment of ECs with sildenafil following Akt3 knockdown reduced expression of these UPRmt-responsive genes to control levels (Fig. 3*B*).

To determine whether reductions in angiogenesis because of Akt3 depletion could also be rescued by sildenafil, *in vitro* angiogenesis assays were performed. As shown in Fig. 3*C* and the branch point quantitation in Fig. 3*D*, Akt3 depletion results in a 5-fold decrease in angiogenesis that is partially rescued by sildenafil treatment. Taken together, these data provide evidence that inhibition of PDE5 rescues mitochondrial function and homeostasis and partially the angiogenic defects following Akt3 depletion and suggests that sildenafil induces an Akt3-independent pathway leading to mitochondrial biogenesis and homeostasis.

### Pharmacological induction of mitochondrial dysfunction is rescued by sildenafil in ECs

**Figure 4:**
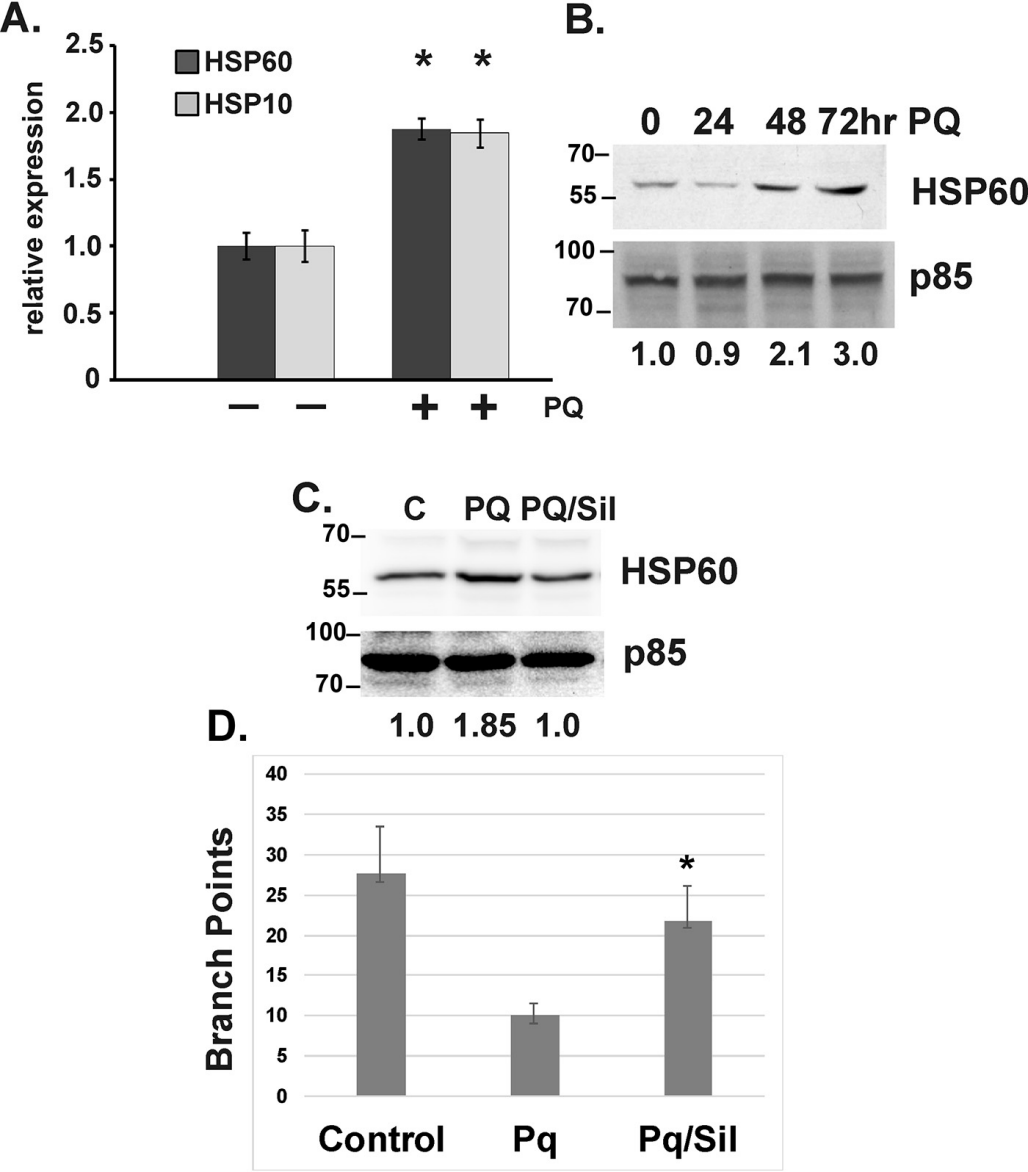
**Pharmacological induction of mitochondrial dysfunction is inhibited by sildenafil.***A*, real-time PCR of total RNA isolated from ECs treated with paraquat (100 nm) for 48 h using primers directed against HSP10 and HSP60. *B*, cells treated similarly to (*A*) and total protein isolated and tested for HSP60 expression by immunoblot in a time course analysis. *C*, Immunoblot of HSP60 using total protein isolated from ECs treated with vehicle control (*C*), paraquat (*PQ*) or paraquat plus sildenafil (*PQ/Sil*). Branch point quantitation of cells subjected to treatment with or without paraquat plus or minus sildenafil (100 μm). *D*, Branch point quantitataion of cells. *, *p* < 0.05 relative to respective control. *Error bars* indicate S.E.

Previous studies have shown that low concentrations of the model oxidant paraquat, which inhibits mitochondrial function and induces oxidative stress, can induce a UPRmt (22, 23, 24). To directly test the effect of sildenafil on pharmacologically induced mitochondrial dysfunction, we treated ECs with paraquat and assessed its effect on chaperone expression. Paraquat treatment results in an increase in the expression of both HSP10 and HSP60 on the mRNA level (Fig. 4*A*) and an increased HSP60 protein expression (Fig. 4*B*). Treatment with sildenafil partially reduces the expression of HSP60 (Fig. 4*C*). Importantly, paraquat decreased branch point and sildenafil partially or completely restored branch point formation, rescuing the angiogenic response (Fig. 4*D*).

### Sildenafil-dependent mitochondrial biogenesis is Akt3-independent and requires PGC-1–related coactivator in ECs

**Figure 5:**
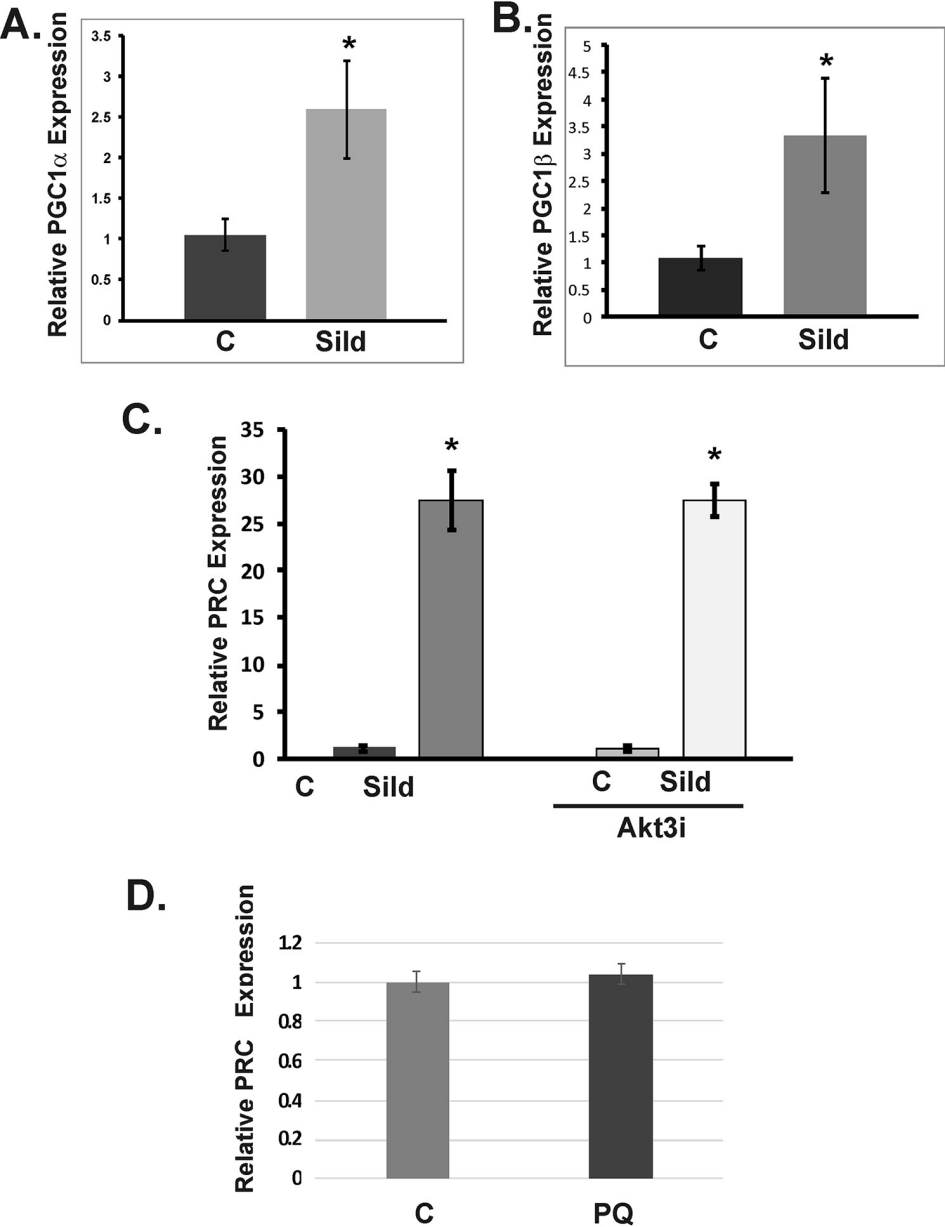
**Sildenafil induces the expression of PRC expression independently of Akt3 depletion.***A* and *B*, real-time PCR of total RNA isolated from ECs treated with or without sildenafil (100 μm) using primers against PGC-1α (*A*) or PGC-1β (*B*). *C*, cells treated as in (*A*) with or without transfection using RNAi directed against Akt3 using primers against PRC in real-time PCR. *D*, ECs treated with paraquat (100 nm) and total RNA used in real-time PCR with primers directed against PRC. All PCR is expressed as relative to S26 as an internal control. *p* < 0.05 for all real-time PCR. *Error bars* indicate S.E.

PGC-1α, PGC-1β, and PRC have both independent and overlapping functions. To determine whether sildenafil affected the expression of this family of transcriptional coactivators, we performed quantitative real-time PCR. Sildenafil treatment increased PGC-1α and PGC-1β expression by ∼2.6- and 3.3-fold, respectively, compared with vehicle control (Fig. 5, *A* and *B*). Sildenafil treatment had the most striking impact on PRC mRNA, which increased over 25-fold compared with vehicle control (Fig. 5*C*). Akt3 depletion had no effect on PRC expression was similar between vehicle control groups transfected with SCR and Akt3 RNAi, and the relative increases in PRC expression following sildenafil treatment were also similar (Fig. 5*C*). PRC expression was not affected by paraquat treatment (Fig. 5*D*). Together, sildenafil induces PGC-1α, PGC-1β, and PRC expression, and this could be alternative pathways to induce mitochondrial biogenesis in ECs in an Akt3-independent manner.

**Figure 6:**
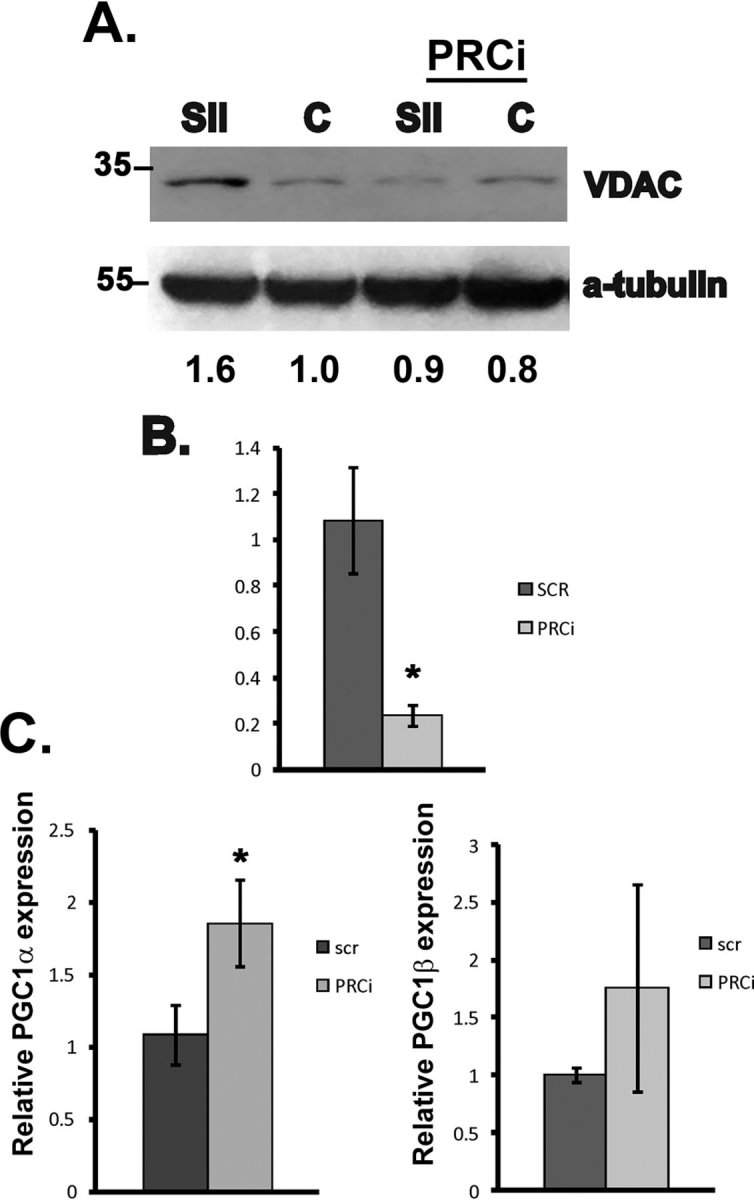
**Sildenafil-dependent mitochondrial biogenesis requires PRC activity.***A*, immunoblot for VDAC protein expression in ECs transfected with siRNA again PRC (*PRCi*) or scramble control (*SCR*) and treated with vehicle or sildenafil (*Sil*) for 72 h. α-Tubulin is shown as a loading control. *B*, real-time PCR for PRC expression in ECs following PRC knockdown by siRNA. *C* and *D*, real-time PCR for PGC-1α (*C*) and PGC-1β (*D*) mRNA expression following PRC knockdown by siRNA. Expression is shown as relative to S26. *Asterisk* indicates significantly different from control (*p* < 0.05).

To determine whether PRC is required for sildenafil-induced mitochondrial biogenesis, we evaluated VDAC expression by Western blotting in ECs following PRC knockdown by siRNA. As shown in Fig. 6*A*, knockdown of PRC blocked sildenafil-dependent increases in VDAC expression, suggesting PRC is required for sildenafil-dependent mitochondrial biogenesis. PRC knockdown was confirmed by real-time PCR (Fig. 6*B***)**. To confirm that the blockade of the sildenafil-dependent increase in VDAC expression following PRC knockdown was specific to loss of PRC expression, we determined the effect of PRC knockdown on PGC-1α and PGC-1β. PRC knockdown increased PGC-1α mRNA expression 1.8-fold. However, PRC knockdown did not result in a statistically significant change in PGC-1β mRNA expression (Fig. 6*C*, *right*).

### Sildenafil promotes the expression of PRC through the cAMP and rescues mitochondrial dysfunction in response to Akt3 depletion

**Figure 7:**
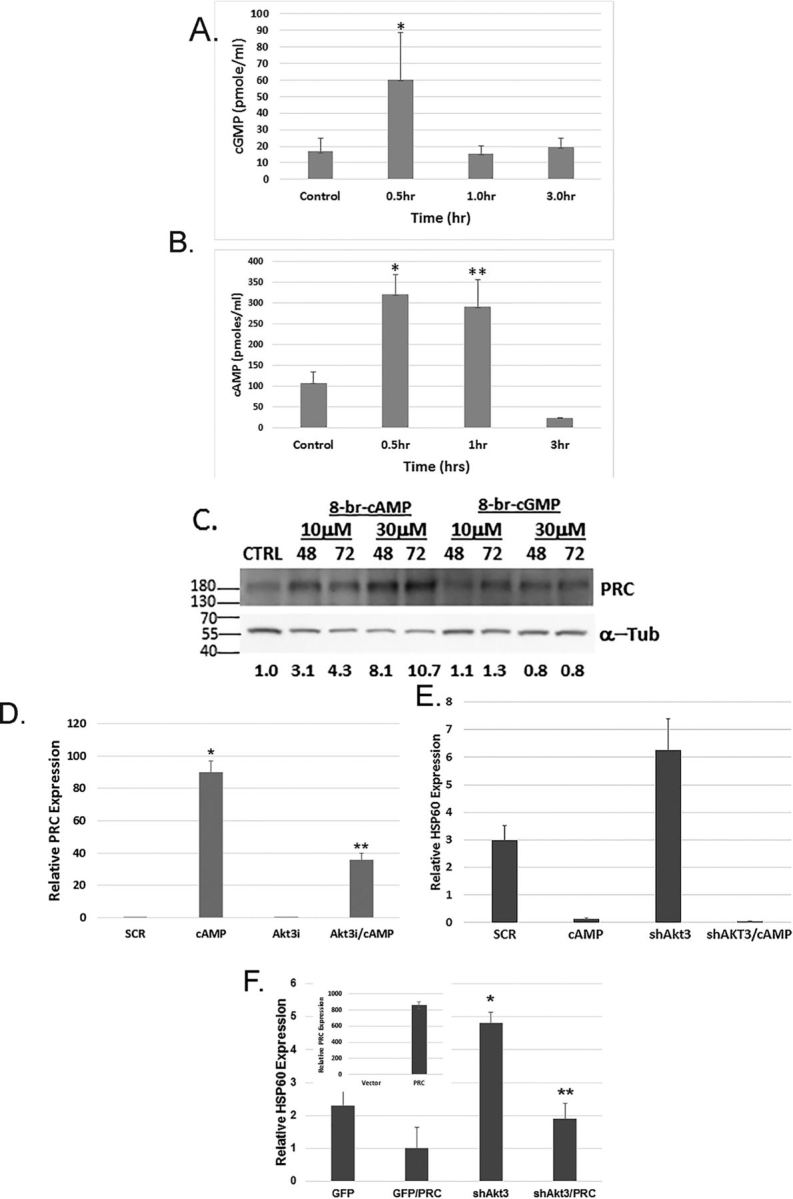
**PRC rescues mitochondrial dysfunction driven by Akt3 depletion.** Sildenafil increases PRC expression via CREB. *A*, quantitation of cGMP amounts after treatment with sildenafil (100 μm). *p* < 0.05*. *B*, quantitation of cAMP amounts after treatment with sildenafil (100 μm). *p* < 0.01 * and ** *p* < 0.001 *C*, Western blot analysis of PRC expression following treatment with two dosages (10 and 30 μm) of 8-bromo-cAMP or 8-bromo-cyclic GMP for 48 and 72 h. Tubulin is shown as an internal control. *D*, real-time PCR of PRC expression in ECs transfected with scramble control (*SCR*) or shRNA directed against Akt3 plus and minus the addition of 8-bromo-cAMP (30 μm) for 48 h. *p* < 0.0.1 * and ** *p* < 0.05. *E*, cells treated as in *D* and assessed for HSP60 expression. *p* < 0.01* and **p < 0.05. *F*, real-time PCR of HSP60 expression following transduction with an shRNA directed against GFP as a control or Akt3 plus and minus transfection with a full-length PRC mammalian expression vector. The *inset* shows a quantitation of PRC overexpression by real-time PCR.

Sildenafil inhibits PDE5, a specific phosphodiesterase that controls cGMP turnover. However, others have shown a feedback loop that also affects cAMP turnover (25). To test whether cGMP or cAMP was required for the increased expression of PRC in response to sildenafil, amounts of cGMP and cAMP after treatment with sildenafil were determined. As shown in Fig. 7*A* (cGMP) and Fig. 7*B* (cAMP), sildenafil causes an increase in the amounts of both cyclic nucleotides, but the increase in cAMP is larger in magnitude and in duration. To test which of the cyclic nucleotides could affect the expression of PRC, ECs were treated with the cell-permeable cyclic nucleotide analogues; 8-bromo-cAMP and 8-bromo-cGMP were used. As shown in Fig. 7*C*, 8-bromo-cAMP treatment causes a 3- to 10-fold increase in PRC expression depending on the quantity and time of treatment. To test whether cAMP could increase PRC expression under conditions of Akt3 depletion, cells were treated with 8-bromo-cAMP and assessed for the expression of PRC by real-time PCR. As shown in Fig. 7*D*, 8-bromo-cAMP caused an increase in the expression of PRC under conditions of Akt3 depletion. To test the hypothesis that PRC is required to rescue the Akt3-dependent mitochondrial dysfunction, PRC was overexpressed in cells either transduced with a scramble control or an shRNA directed against Akt3 and tested for changes in HSP60, a marker of mitochondrial dysfunction. Fig. 7*E* shows that HSP60 expression is rescued by inclusion of cAMP under conditions of Akt3 depletion. Fig. 7*F* shows that PRC overexpression reduces the expression of HSP60 under conditions of Akt3 depletion.

### Sildenafil promotes the expression of PRC through the cAMP-responsive element binding protein CREB

**Figure 8:**
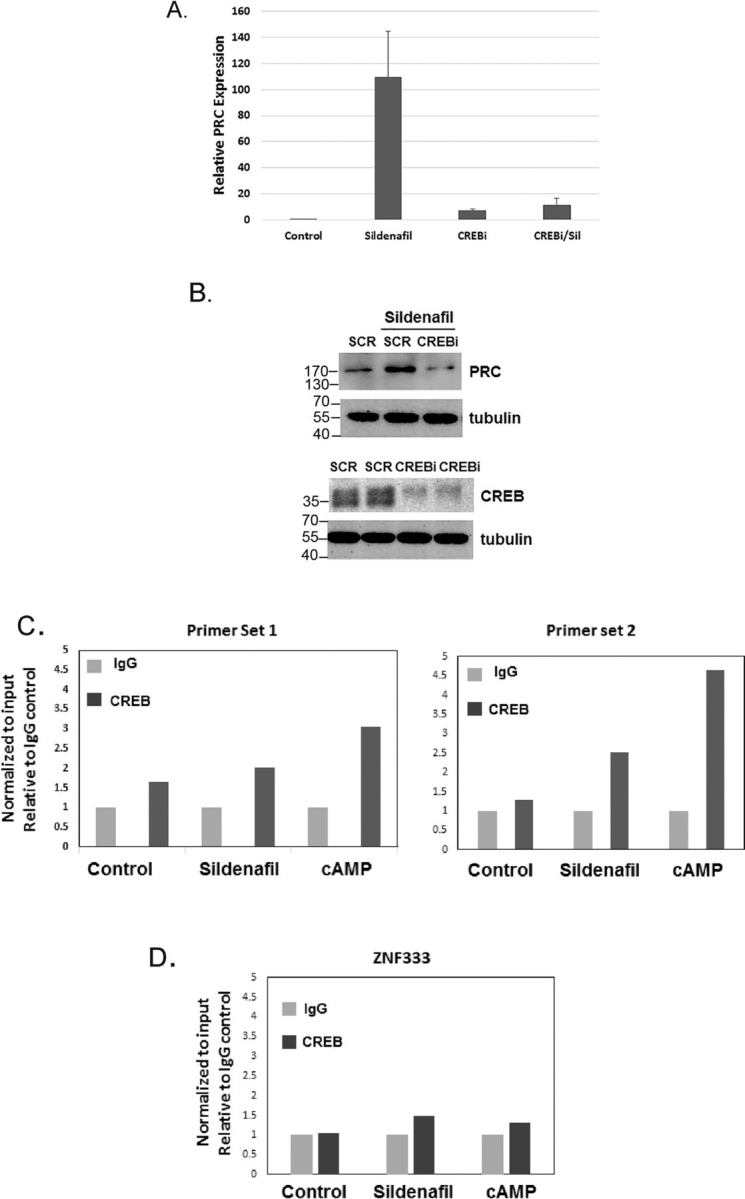
*A*, real-time PCR of PRC expression in ECs transfected with scramble control (*Control*) or an RNAi directed against CREB and left untreated. *B*, Western blot analysis of ECs treated as in *D* (*upper panel*). *Lower panel* shows efficacy of CREB knockdown by Western blot analysis. Representative experiments are shown from an *n* = 3. *C*, CHIP analysis of CREB using two sets of primers plus and minus sildenafil or bromo-8-cAMP (30 μm) for 24 h. Primer set 1 (*n* = 1); primer set 2 (*n* = 2). Data are shown normalized to input and relative to IgG control. *D*, CHIP analysis of CREB using primers directed against ZNF333 as a nonspecific control.

Because cAMP causes an increased expression of PRC and that PRC expression can be controlled by the cAMP-responsive element binding protein CREB, we tested whether CREB depletion would affect the sildenafil-dependent expression of PRC. As shown in Fig. 8*A*, real-time PCR of PRC expression induced by sildenafil is blocked by CREB depletion. The Western blotting in Fig. 8*B* confirms the requirement of CREB expression for the induction of PRC by sildenafil. To determine whether CREB was directly modulating PRC transcription, we sought to determine whether CREB specifically bound to the PRC promoter region. *In silico* ChIP-Seq analysis suggested the presence of a potential CREB binding site overlapping exon 1 of PRC. This site had been positively identified as CREB-binding sites by ChIP-Seq in several other cell types (RRID:SCR_015482). Using two primer sets directed against this potential CREB site, we find that each primer set resulted in increased CREB binding to the PRC promoter, as compared with IgG control, in response to either sildenafil or 8-bromo-cAMP (Fig. 8*C*). Primers directed against the zinc finger gene, ZNF333 were used as a negative control (Fig. 8*D*).

## Discussion

**Figure 9:**
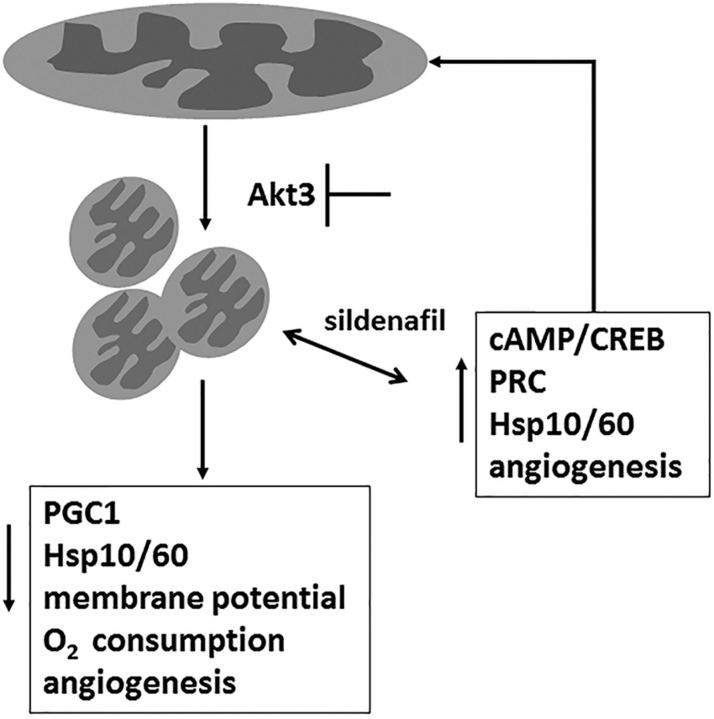
**A working model of Akt3-dependent regulation of mitochondrial homeostasis and angiogenesis.** Mitochondrial stress induced by either Akt3 depletion or pharmacological inhibition increases the expression of the mtUPR genes HSP10 and HSP60 and blocks angiogenesis. Sildenafil through increased PRC expression rescues mitochondrial stress, increases mitochondrial biogenesis and allows for angiogenic responses.

Our results support the hypothesis that Akt3-dependent mitochondrial biogenesis is required for angiogenesis and suggest the working model in Fig. 9 Mitochondrial stress induced by Akt3 depletion or pharmacologically by model oxidant paraquat results in decreased mitochondrial function, increased expression of the UPRmt chaperones HSP10 and HSP60, and a reduction in angiogenesis. Increased cAMP signaling by sildenafil rescues this phenotype by reducing the expression of HSP10 and HSP60 while increasing mitochondrial biogenesis via the expression of PRC. We find that PRC expression is controlled by CREB, which specifically binds just 5′ to the first exon of PRC. The resultant decreased mitochondrial stress by sildenafil restores the angiogenic potential of endothelial cells in an Akt3-independent manner. Akt3 controls transcription of mitochondrial biogenesis genes via the nuclear retention of PGC-1α. Akt3 depletion results in a decrease in PGC-1α–dependent transcription of numerous proteins, a marked reduction in mitochondrial biogenesis, and increased autophagy. Because of either direct decreases in mitochondrial protein import efficiency or general decreases in mitochondrial protein production, perturbation of the Akt3/PGC-1α pathway or inhibition of electron transport by paraquat causes mitochondrial protein stress and ultimately mitochondrial dysfunction leading to decreased angiogenesis. Increasing cAMP by blockade of PDE5 with sildenafil rescues mitochondrial stress by increasing the expression of PRC via direct binding of CREB to the PRC promoter, increasing mitochondrial biogenesis leading to increased angiogenesis. cAMP has been shown to be a key driver of angiogenesis downstream of vascular endothelial growth factor signaling (26).

Our findings show that sildenafil, although known as a PDE5 inhibitor decreasing the turnover of cGMP, also results in an increase in cAMP. Others have also shown that sildenafil treatment can increase cAMP in EC and other cell types (27, 28, 29). It is thought that this increased cAMP is caused by a feedback loop affecting other phosphodiesterases that control cAMP. The requirement of cAMP for induction of PRC expression was confirmed using the cell-permeable compound 8-bromo-cAMP.

We have shown that Akt3 is required for angiogenesis and that overall mitochondrial health (*i.e.* respiratory capacity) is a prerequisite for launching angiogenic responses. The fact that a mitochondrial biogenic compound that inhibits PDE5 and increases cAMP to produce mitochondrial biogenesis can bypass the Akt3 pathway suggests redundancy in pathways capable of restoring mitochondrial homeostasis. It is well understood that multiple pathways converge on the regulation of mitochondrial biogenesis through PGC-1α, including, ROS, cAMP, cGMP, and p38 (30). However, here we provide evidence that activation of PRC expression by sildenafil is sufficient to restore mitochondrial function following Akt3 knockdown. As cGMP can activate eNOS, it is possible that NO, which also activates the PGC-1α transcriptional program, increases the expression of all three PGC-1 family members, with greatest impact on PRC expression, to functionally bypass Akt3 blockade and increasing mitochondrial biogenesis to restore mitochondrial health. Akt3 is also required for mitochondrial function in cancer cells (31). The exact mechanism by which specifically Akt3 and not Akt1/2 affects mitochondrial homeostasis and the role of the PGC-1 and PRC in this response is presently under investigation.

Others have reported that PGC-1α is a negative regulator of angiogenesis in the endothelium. Vascular specific ablation of PGC-1α results in a marked increase in angiogenesis *in vivo* and *in vitro* (32). These findings suggest that in addition to its regulation of mitochondrial biogenesis, PGC-1α also regulates angiogenic potential. Interestingly, in data not shown, we see that depletion of either PGC-1α or PRC in ECs results in a marked increase in angiogenesis in Matrigel assays. This effect overrides any positive effect of sildenafil on angiogenesis. We were therefore unable to test whether PRC was required for angiogenesis downstream of sildenafil. It is interesting to note that PGC-1 or PRC knockouts have no overt mitochondrial dysfunction at baseline, suggesting redundancy in the mechanisms controlling mitochondrial homeostasis. The molecular mechanism by which these transcriptional co-activators negatively regulate angiogenesis and whether this activity is independent of their regulation of mitochondrial biogenesis is currently under investigation in the laboratory.

There is an open question as to the exact role of the mitochondria during angiogenesis. A recent study from De Bock *et al.* (33) suggests that ATP supplied by the mitochondrial oxidative phosphorylation is not required to launch an angiogenic response, as inhibition or augmentation of mitochondrial respiration had no effect on angiogenic responses *in vitro*. Instead, their study suggests that glycolysis is responsible for the energetic homeostasis of ECs and that ablation of phosphofructokinase-2/fructose-2,6-bisphosphatase 3 (PFKFB3), a key glycolytic enzyme, can halt angiogenesis altogether (33). However, it is important to note that De Bock *et al.* (33) used oligomycin and antimycin A as inhibitors of mitochondrial electron transport chain (ETC). Oligomycin is a selective inhibitor of ATP synthase. Inhibition of ATP synthase would likely result in hyperpolarization of mitochondria, as electron transport through complexes I–IV can continue even though mitochondrial ATP production is blocked. Hyperpolarization of the mitochondria is known to increase ROS production (34), which is known to initiate angiogenesis. The second ETC inhibitor used in this study, antimycin A, inhibits electron flow through ETC complex III and is known to produce superoxide as a result (35). Interestingly, De Bock *et al.* (33) showed increased *in vitro* angiogenic responses following antimycin A treatment. Although this study likely shows mitochondrial ATP production is not required for angiogenesis, there may be a requirement for mitochondrial ROS.

Counter to the findings from De Bock *et al*. (33), a study from Dranka *et al*. (36) found that cellular ATP levels within ECs were directly linked with mitochondrial respiratory capacity, suggesting differential pathways for ATP production could be employed as cellular demand changes (1). Here, an angiogenic compound increases the maximal respiratory capacity without effect on basal oxygen consumption, suggesting a possible link between EC respiratory capacity and angiogenic potential. Taken together with the findings of Dranka *et al*. (36), our data suggest mitochondrial respiration may serve as a critical primary/alternative metabolic pathway during angiogenesis or potentially allow for adaptation to stresses, such as oxidative stress or altered calcium signaling (1, 37). Indeed, the presence of high mitochondrial reserve capacity has been shown to positively correlate with stress tolerance (38). ECs, regardless of treatment group, can up-regulate glycolysis following treatment with oligomycin (data not shown), which would further suggest the requirement of mitochondrial pro-angiogenic ROS signaling and calcium buffering.

Mitochondria contain over 1000 proteins, only 13 of which are encoded by mitochondrial DNA. There is tight control of import and folding of nascent proteins. The UPRmt is a transcriptional regulatory program that is induced in response to perturbations in proteostasis. The UPRmt joins other mitochondrial regulatory pathways that are induced upon stress or mitochondrial dysfunction such as autophagy, biogenesis, and fission/fusion, processes in place to ensure mitochondrial health and function. Akt3 depletion results in the down-regulation of the PGC-1α–dependent gene TOM70 (11), the major import protein of the mitochondria, in addition to the induction of heat shock proteins HSP10 and HSP60, decreased mitochondrial biogenesis, and increased autophagy, suggesting that Akt3 is a key regulator of mitochondrial stress in the vasculature (8). Indeed, Sirt3, which is induced by mitochondrial stress and regulates antioxidant responses through the acetylation of FOXO3A, is also negatively regulated under conditions of Akt3 depletion (8). That sildenafil can mitigate mitochondrial stress caused by Akt3 depletion or paraquat through its up-regulation of PRC suggests that PRC has pleotropic effects, like those described for PGC-1α, that include but are not limited to the control of mitochondrial biogenesis. A complete understanding of how these two family members regulate mitochondrial stress pathways remains to be determined; however, it is likely that they will have both redundant and singular functions.

Mitochondrial biogenesis and quality control are essential to human health. Dysfunction at any single step has implications in multiple pathologies. Indeed, mtDNA mutations are implicated in cancer pathogenesis (39). Impaired mitochondrial proteostasis is implicated in multiple neurological diseases, including Alzheimer's disease (40). Defects in mitochondrial fission/fusion dynamics have been demonstrated to cause dominant optic atrophy, and improper mitochondrial degradation has been implicated in development of Parkinson's disease (41). As the role of the mitochondria in the vasculature is much less clear, studies focusing on this organelle within the vascular endothelium will undoubtedly contribute to the understanding of multiple cardiovascular pathologies, including those implicating oxidative stress, such as hypertension and diabetic vascular dysfunction (42). Our data demonstrate the requirement of the Akt3 in mitochondrial proteostasis in the vascular endothelium, and the requirement of mitochondrial homeostasis for angiogenesis. Indeed, data in this study suggest the Akt3 pathway is required for communication between the mitochondria and nucleus in parallel with the UPRmt pathway to mitigate mitochondrial stress. Future studies will investigate the role of Akt3 as a nodal kinase in the regulation of mitochondrial homeostasis in human disease.

## Experimental procedures

### Cell culture, transfection, and drug treatments

Pooled, multiple donor human umbilical vein cells (EC) (Lonza, Basal, Switzerland) were maintained at 37°C with 5% CO_2_ in endothelial basal medium 2 (Lonza) supplemented with EGM-2 SingleQuots. ECs were transfected using an Amaxa Nucleofection system as described by the manufacturer. Briefly, 2 × 10^6^ cells were transfected per cuvette, using no more than 6 μg of vector per transfection. The efficiencies of all transfections were monitored via GFP expression using a GFP expression vector pGFP-C1 (Clontech, Mountainview, CA) or a GFP-directed RNAi (Amaxa, Gaithersburg, MD). RNAi was purchased from Dharmacon (Akt3) or Santa Cruz Biotechnology (Akt3 and PGC-1α). ECs were treated with sildenafil (Tocris, final concentration 100 nm) for 48–72 h, the concentration known to induce mitochondrial biogenesis in renal epithelial cells (21). EC were treated with 8-bromo-cAMP or 8-bromo-cGMP (Sigma) at 10 or 30 μm for 24 h.

### Real-time PCR analysis

cDNA was synthesized from 2 μg of total RNA with a SuperScript First-Strand Synthesis Kit purchased from Invitrogen, using oligo(dT) according to the manufacturer's instructions. Real-time PCR was performed using a Light Cycler 480, from Roche Diagnostics. For each experiment, *n* ≥ 3 for each respective group and was performed at least in triplicate. Primer sequences are as follows (written 5′ to 3′). PGC-1α forward, GCAGAGAGGGAACTTTGCAC, reverse, AGGGCTCAACTAATCGCTCA; PGC-1β forward, GATGTCAGCAAACCCTACC, reverse, TCTTCCTCCTCCTCTTCTTC; PRC forward, CTCAGGGTTCTGAAGATGTG, reverse, CGGTGGACTTAGGAGATTTG; HSP60 forward, TGAAGGTTGGTGGGACAAGT, reverse, ATGAGTCCAAGGCTGGAATG; HSP10 forward, ATGGCAGGACAAGCGTTTAG, reverse, CAGCGACTACTGTTGCTTGC; and S26 forward, CTCCGGTCCGTGCCTCCAAG, reverse, CAGAGAATAGCCTGTCTTCAG.

### Western blot analysis

Antibodies used for Western blot analysis are PRC (Abcam), VDAC (Santa Cruz Biotechnology), and α-tubulin (Sigma). Appropriate HRP-conjugated secondary antibodies were purchased from Invitrogen. Treated cells were washed once with PBS and lysed in 1× RIPA Lysis Buffer (50 mm Tris-HCl, pH 7.5, 1% Triton X-100, 150 mm NaCl, 0.1% SDS, 1% sodium deoxycholate, 40 mm NaF), supplemented with complete protease inhibitors without EDTA (Roche) and 200 μm sodium orthovanadate. Protein concentrations were measured using the BCA protein assay (Pierce, Rockford, IL), resolved by SDS-PAGE, and transferred onto Immobilon-P PVDF membranes (Millipore). Western blots were visualized with luminol reagent (Santa Cruz Biotechnology).

### Immunofluorescence analysis

Antibody used for immunofluorescence was anti-GFP (Molecular Probes). In addition, MitoTracker Deep Red (Life Technologies) was used to stain mitochondria. All fluorescent-tagged secondary antibodies were purchased from Molecular Probes, Invitrogen. Transfected or treated ECs were seeded onto poly-lysine–coated coverslips, fixed in 3.7% formaldehyde for 20 min, and washed briefly in 1× PBS. Cells were permeabilized in 0.1% Triton 100-X for 20 min, washed briefly in 1× PBS, and blocked for 30 min in 5% BSA-PBS under gentle agitation. Cells were subsequently incubated in primary antibody for 1 h at room temperature, and then washed in 1× PBS and incubated with the appropriate fluorescent secondary antibody for 1 h at room temperature. After washing in 1× PBS, cell nuclei were stained with DAPI nuclear dye (Molecular Probes) and coverslips were mounted to slides using Fluorogel (Electron Microscopy Sciences, Hatfield, PA). Coverslips were imaged on a Zeiss Axio Imager M2 fluorescent microscope.

### Matrigel angiogenesis assay

ECs were transfected with scrambled (control) RNAi, Akt3 RNAi or treated with paraquat (10 μm) prior to plating onto Matrigel matrix (BD Laboratory). Equal cell numbers were used for each assay, which was repeated at least in triplicate. For assay quantitation, branch points were counted from six fields per well (at 20× magnification), with three wells per assay.

### Oxygen consumption analysis

EC oxygen consumption was measured using an XF96 Extracellular Flux Analyzer (Seahorse Biosciences). Cells were seeded in a 96-well plate (final density ∼20,000 cells/per well). After baseline measurements, HUVECs were challenged to a mitochondrial stress test via sequential administration of oligomycin followed by FCCP.

### Quantitation of cAMP and cGMP

Competitive ELISA analyses (Cayman Chemical) were used to quantitate cAMP or cGMP concentrations in cells treated with sildenafil (100 nm) in times indicated in the text. Assays were performed as described by the manufacturer in at least quadruplicate, three independent times.

### Statistics

For comparisons between two groups, a Student's *t* test was used. For comparisons of three or more groups, an analysis of variance was performed, followed by a Newman-Keuls post hoc analysis for direct comparisons among individual groups. Because the exact number of cells exhibiting decreased Akt3 expression is unknown, the relative risk of fragmentation (probability of fragmentation comparing control *versus* Akt3 depletion) was calculated.

### Chromosome immunoprecipitation

HUVEC cells were crosslinked with 1% formaldehyde for 10 min at room temperature. The reaction was quenched with 125 mm glycine for 5 min at room temperature. Crosslinked cells were washed twice with ice-cold PBS, scraped into PBS containing HALT protease and phosphatase inhibitors (Thermo Fisher/Pierce) and pelleted. Chromatin was isolated using MNase enzymatic digestion following the manufacturer's protocol (SimpleChIP® Enzymatic Chromatin IP Kit, Cell Signaling Technologies). Briefly, MNase was added to isolated nuclei at optimized concentrations. The digestion reaction mix was incubated at 37°C for 20 min. EDTA was then added to stop the reaction. The MNase-digested chromatin was released from the nuclei by brief sonication. Digested chromatin was precleared with protein G beads for 2 h at 4°C. Equal amounts of pre-cleared chromatin were incubated with CREB (Cell Signaling Technology) or normal IgG (Cell Signaling Technology) primary rabbit antibodies overnight at 4°C. The following day, protein G magnetic beads were added to the sample-antibody mixture and incubated at 4°C for 3 h. Beads were then washed three times in a low-salt wash buffer, and once in a high-salt wash buffer. Samples were reverse crosslinked in elution buffer at 65°C. Reverse crosslinked samples were subjected to proteinase K digestion and the DNA isolated using DNA-binding columns. ChIP-rPCR analysis was completed with the appropriate primer sets (Integrated DNA Technologies, Coralville, IA, USA) using Power SYBR Green PCR assay systems (Thermo Fisher/Applied Biosystems) using a Bio-Rad CFX384 real-time PCR machine. Primers are as follows: ZNF333, forward, 5′ TGC AGC CAG TGT GGG AAA GC 3′, reverse, 5′ GTG CTC GTC CGG AAG GGC TTG 3′; PRC Primer set 1, forward, 5′ AGG TGA GGA TTA GCG CTT GG 3′, reverse, 5′ TGA CGT TCT ACC TGC TGT ACG 3′; and PRC Primer set 2, forward, 5′ TGC GTT ACA CTG GGA TAC CG 3′, reverse, 5′ TGC GTT ACA CTG GGA TAC CG 3′.

## Data availability

Branch point analysis of PRC and PGC-1 knockdown and basal respiration rates are available upon request (Robin Muise-Helmericks, Medical University of South Carolina, musehelm@musc.edu. All other data are presented in the manuscript.

10.13039/100000050HHS | NIH | National Heart, Lung, and Blood Institute (NHLBI) (2T32HL007260-36) to Daniel G. Corum, and Robin C. Muise-Helmericks
10.13039/100000050HHS | NIH | National Heart, Lung, and Blood Institute (NHLBI) (HL084565) to Daniel G. Corum, and Robin C. Muise-Helmericks
